# Temporal trends and treatment patterns in anal fissure management: insights from a multicenter study in Italy

**DOI:** 10.1007/s10151-024-03011-4

**Published:** 2024-10-04

**Authors:** A. Picciariello, R. Tutino, G. Gallo, D. F. Altomare, R. Pietroletti, A. Dezi, G. Graziano, SICCR Anal Fissure Group, Ambrosini Fabio, Annicchiarico Alfredo, Antonacci Nicola, Ascari Francesca, Ascenzi Pasquale, Balla Andrea, Barugola Giuliano, Basso Luigi, Beati Claudio, Bellato Vittoria, Benatti Emanuela, Bertoli Paolo, Bottini Corrado, Bracchitta Salvatore, Cafaro Danilo, Calussi Marco, Caminati Filippo, Candilio Giuseppe, Cantarella Salvatore, Carbone Fabio, Carini Stefano, Carrino Francesco, Cestaro Giovanni, Chessa Antonella, Ciferri Enrico, Clementi Marco, Coco Claudio, Cocorullo Gianfranco, Colombo Francesco, Comba Andrea, Conti Luigi, Cracco Nicola, Cravero Francesca, Crea Nicola, Crescenti Fabio, Cuccomarino Salvatore, D’Acapito Fabrizio, D’Onghia Giuliano, De Rosa Michele, Di Pietrantonio Daniela, Dodi Giuseppe, Ferrario Luca, Fontana Tommaso, Foti Nicola, Geremia Carmelo, Giannini Ivana, Giordano Pasquale, Giuliani Antonio, Guaitoli Eleonora, Laforgia Rita, Lantone Giuliano, Lemma Maria, Lisi Giorgio, Lobascio Pierluigi, Lovisetto Federico, Lucci Enrico, Madeddu Francesco, Magnani Costantino, Mascali Davide, Merlini David, Milito Giovanni, Miro Antonio, Moggia Elisabetta, Monaci Iacopo, Mozzon Marta, Navarra Luca, Oggianu Angelo, Orlandi Simone, Palumbo Alessio, Passaro Umberto, Pata Francesco, Pecorella Giuseppe, Pedrazzani Corrado, Piccolo Davide, Poli Giulia, Rinaldi Marcella, Ripetti Valter, Rizzo Salvatore, Rocco Giuseppe, Sacco Michele, Saroglia Giuliano, Scotto Bruno, Selvaggi Lucio, Silvestri Vania, Soldini Gabriele, Tamini Nicolò, Tanda Cinzia, Terrosu Giovanni, Testa Alessandro, Tomasicchio Giovanni, Turati Luca, Ursino Natale, Vannelli Alberto, Viola Gabriele, Violante Tommaso, Zigiotto Daniele, U. Grossi

**Affiliations:** 1 Department of Experimental Medicine, University of Salento, Lecce, Italy; 2 Department of General and Emergency Surgery, AOU Città della Salute e della Scienza, Turin, Italy; 3 Department of Surgery, Sapienza University of Rome, Rome, Italy; 4 Department of Precision and Regenerative Medicine and Ionian Area and Interdepartmental Research Center for Pelvic Floor Diseases (CIRPAP), University Aldo Moro of Bari, Bari, Italy; 5 Department of Biotechnological and Applied Clinical Sciences, Proctology Unit, University of L’Aquila, L’Aquila, Italy; 6 Center for Outcomes Research and Clinical Epidemiology (CORESEARCH), Pescara, Italy; 7 Oncology and Gastroenterology – DiSCOG, University of Padova, Padua, Italy

**Keywords:** Anal fissure, Botulinum toxin injection, Conservative treatment, Surgery, Trends, Sphincterotomy, Fissurectomy

## Abstract

**Introduction:**

Anal fissure (AF) poses a common challenge in clinical practice, prompting various treatment approaches. This multicenter study, conducted by the Italian Society of Colorectal Surgery, aimed to assess treatment trends in AF over a 10 year period.

**Methods:**

A survey of proctologists and retrospective analysis of patient records were conducted to evaluate treatment modalities and outcomes across six different clinical scenarios based on AF presentation (acute/chronic) stratified by sphincter function (normal/hypertonic/hypotonic).

**Results:**

Analysis of data from 17 principal investigators and 22,016 patients revealed significant variability in treatment approaches, influenced by factors such as symptom duration, anal tone, and surgeon preference. Conservative treatments were commonly utilized, while surgical interventions were reserved for refractory cases. Specifically, pharmaceutical treatment was administered to 66–75% of patients in cases of acute AF and 63–67% for chronic AF, while 10–15% underwent anal dilation, and < 2% received botulinum toxin injection. Among medical treatments, nifedipine with lidocaine and glycerin film-forming ointments were the most utilized. The most performed surgical techniques were fissurectomy and anoplasty, except for patients with chronic AF and hypertonic sphincter where sphincterotomy prevailed. Trends in treatment utilization varied depending on the clinical scenario, with notable shifts observed over time.

**Conclusions:**

This study provides insights into the evolving landscape of AF management, highlighting the need for further research to elucidate optimal treatment strategies and improve patient outcomes.

**Supplementary Information:**

The online version contains supplementary material available at 10.1007/s10151-024-03011-4.

## Introduction

Anal fissure (AF) is one the most common and painful conditions affecting the anal region, often leading patients to seek care from proctology units. It significantly impacts patients’ quality of life and imposes a considerable burden on the public health system [1].

While some cases may resolve spontaneously, AF frequently persists despite standard medical treatments, becoming a chronic condition [2].

Diagnosing AF typically involves a proctological examination, with hypertonicity of the internal anal sphincter being a common finding. However, normal or even reduced anal tone can also be observed, necessitating different treatment strategies.

Various medical treatments have been proposed, with calcium-channel blockers, nitroglycerin, topical anesthetics, and glycerin film-forming ointments among the options [3]. The optimal duration of these treatments remains unclear and is usually based on symptom relief and fissure healing time.

When conservative treatments prove ineffective, surgical intervention may be necessary. Available surgical options aim to reduce the anal sphincter tone [such as internal/external sphincterotomy and botulinum toxin (BTX) injection] or directly address the fissure through fissurectomy, with possible repair of the anal canal (anoplasty) [4, 5].

This multicenter study aims to assess changes in treatment trends, both conservative and surgical, over a 10 year period.

## Methods

Surgeons affiliated with the Italian Society of Colorectal Surgery (SICCR) were invited to participate in a survey exploring the management of AF. The survey, developed by a consensus of surgeons with expertise and publications in this field, consisted of an online questionnaire. Eleven questions written in English were developed, covering participants’ demographics, diagnostic procedures, and nonoperative and operative treatments. Completion of all questions was mandatory. After testing its functionality, the questionnaire was administered using Google Forms.

Centers handling a minimum of 50 cases of AF annually were then requested to provide data via an Excel spreadsheet, documenting the number and types of procedures performed for AF each year from 1 January 2013 to 31 December 31 2022. Six clinical scenarios of AF based on possible clinical features were proposed, detailing disease duration (acute or chronic, with the latter defined by a fissure persisting beyond 6 weeks [2]) and anal sphincter tone (normal, hypertonic, hypotonic). For each clinical scenario, three medical treatments (glycerin film-forming ointments, nifedipine with lidocaine, and nitroglycerin) and seven operative/surgical techniques (anal dilation, botulinum toxin injection [alone or in combination with fissurectomy], open internal sphincterotomy, closed internal sphincterotomy, anoplasty ± flap, fissurectomy) were considered. The data were retrospectively obtained from prospectively maintained databases in all centers.

### Statistical analysis

The collected data were reviewed and summarized as percentages, and trends over the study period were analyzed. Univariable multinomial logistic regression models were used to compare multiple groups of procedures and assess their association with time, separately for each subtype of AF. Predicted probabilities derived from the models were also depicted graphically. Statistical analyses were conducted using the Statistical Analysis System (SAS) Package, Release 9.4 (SAS Institute, Cary, NC).

## Results

A total of 115 out of 152 proctologists affiliated with the SICCR, accepted to participate and completed the preliminary questionnaire (Appendix 1), with 17 (14.7%) principal investigators from 17 centers meeting the inclusion criteria (treating at least 50 fissures annually) and, therefore, completing the Excel database.

The preliminary questionnaire revealed that the primary factors guiding treatment selection for patients with AF were symptom duration and anal tone (84.3% of the respondents). Treatment decisions were also influenced by healing rates (43.5% agreement), postoperative incontinence rates (32.2% agreement), and surgeon preference (21.7% agreement). The patient preference was considered by only 2.6% of respondents. Incontinence scores were routinely calculated by 40.9% of proctologists before and after treatments.

Regarding medical treatment, 50.4% of respondents agreed that the choice of ointment administered was influenced by both cost and adverse events. The latter had a greater impact on the choice of ointment. Indeed, only 2.7% believed that the selection was solely based on the cost of the ointment, while 26.5% indicated that adverse events alone played a decisive role.

Data were collected from a total of 22,016 patients across six different scenarios: 3378 in scenario A, 8050 in scenario B, 918 in scenario C, 2599 in scenario D, 6181 in scenario E, and 890 in scenario F.

## Acute anal fissure

### Scenario A—normal sphincter tone

Among 3378 patients in this scenario, conservative treatment was administered to 2386 (70.6%), with glycerin film-forming ointments being the most common [1368 (40.5%) patients]. The use of nifedipine with lidocaine was observed in 24.9% of patients. Anal dilation and BTX injection were performed in 400 (11.8%) and 28 (0.8%) subjects, respectively. Overall, 564 patients (16.7%) underwent surgery, with fissurectomy being the most performed operation (269 cases, 7.9%) followed by anoplasty [248 (7.3%) cases].

**Fig. 1:**
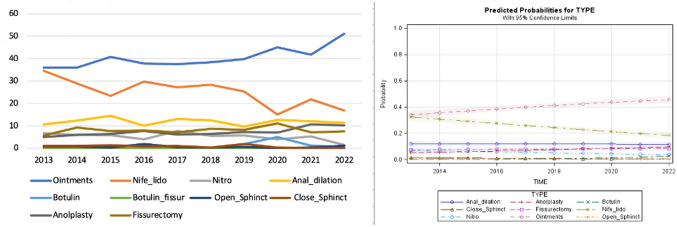
Trend of the different medical and surgical treatments over time and predicted probability model for scenario A (normal sphincter function)

The use of glycerin film-forming ointments increased over time as opposed to nifedipine with lidocaine, while surgical interventions remained limited (Fig. 1).

### Scenario B—hypertonic internal anal sphincter

Out of 8050 patients, conservative therapy was administered in 6055 (75.2%) cases, with anal dilation, yet performed in 1043 (12.9%) patients, showing a decreasing trend over time.

Among conservative treatments, nifedipine with lidocaine was the most administered ointment [2605 (32.3%) patients]. Closed internal sphincterotomy was the most performed operation [279 (3.4%) patients], followed by anoplasty (3.2%), open internal sphincterotomy (3.1%), and fissurectomy (1.6%).

**Fig. 2:**
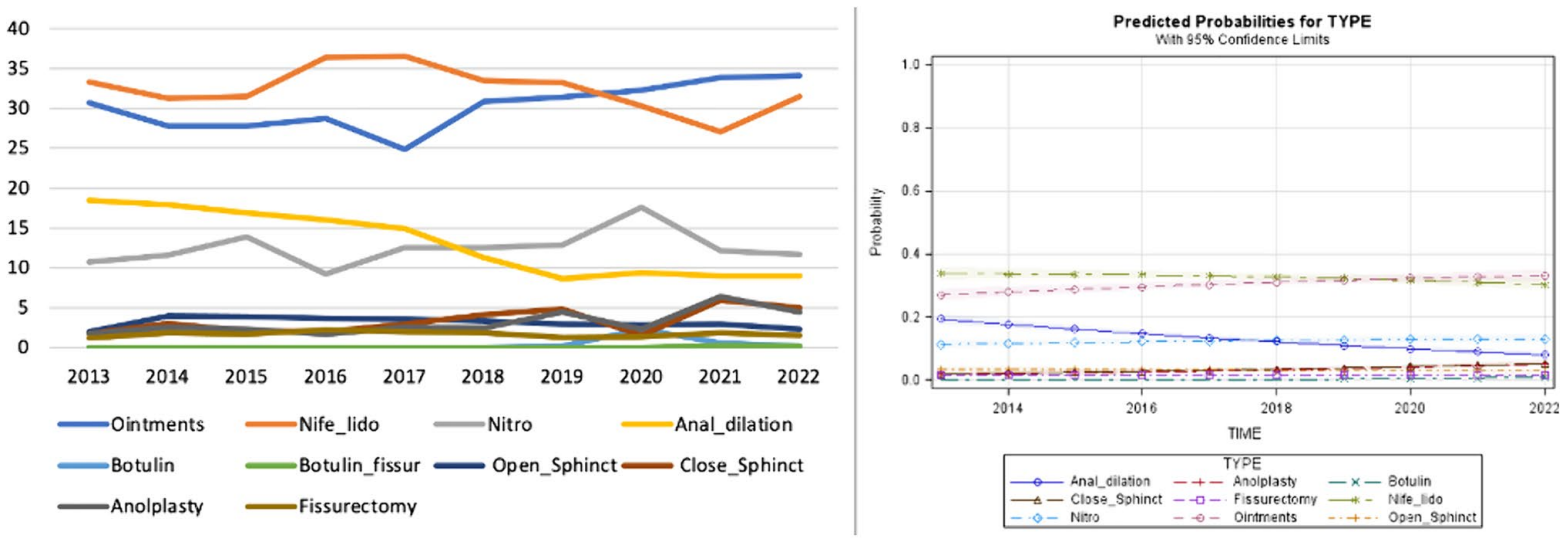
Trend of the different medical and surgical treatments over time and predicted probability model for scenario B (hypertonic internal anal sphincter)

The predicted probability model confirmed the decreasing trend of anal dilation and the increasing trend of conservative treatments. All surgical techniques remained with stable curves over time (Fig. 2).

### Scenario C—hypotonic anal sphincter

**Fig. 3:**
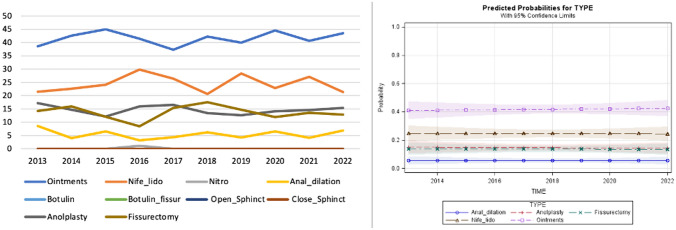
Trend of the different medical and surgical treatments over time and predicted probability model for scenario C (hypotonic anal sphincter)

Of 918 patients, medical treatment was chosen for 609 (66.4%) subjects, with glycerin film-forming ointments [383 (41.7%) patients], and nifedipine with lidocaine [225 (24.5%) patients] being the most common options. Surgical techniques remained stable over time (Fig. 3).

## Chronic anal fissure

### Scenario D—normal sphincter tone

**Fig. 4:**
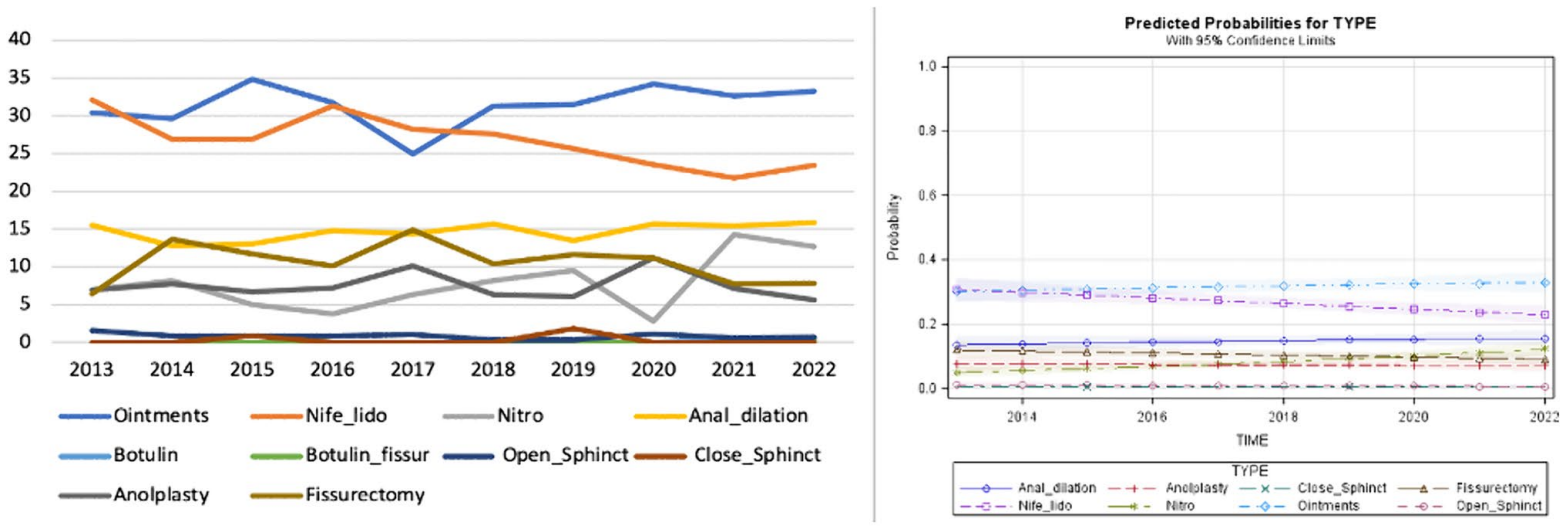
Trend of the different medical and surgical treatments over time and predicted probability model for scenario D (normal sphincter function)

For this scenario, data from 2599 patients were collected. Medical treatment was prescribed for 1731 (66.6%) patients, while surgery was carried out in 483 cases. Anal dilation and BTX injection were performed in 383 and 2 patients, respectively. Following an increasing trend between 2014 and 2016, the administration of nifedipine with lidocaine [682 (26.2%) subjects] slightly decreased over time. Conversely, glycerin film-forming ointments saw an opposite trend, with 825 (31.7%) patients receiving this treatment. Anal dilation was more frequently utilized in the last 4 years. Fissurectomy was the most performed surgical technique [267 (10.3%) patients] followed by anoplasty (7.2%). According to the predicted probability model, a decreasing trend was observed for nifedipine with lidocaine, while an increasing trend was shown for anal dilation (Fig. 4).

### Scenario E—hypertonic internal anal sphincter

**Fig. 5:**
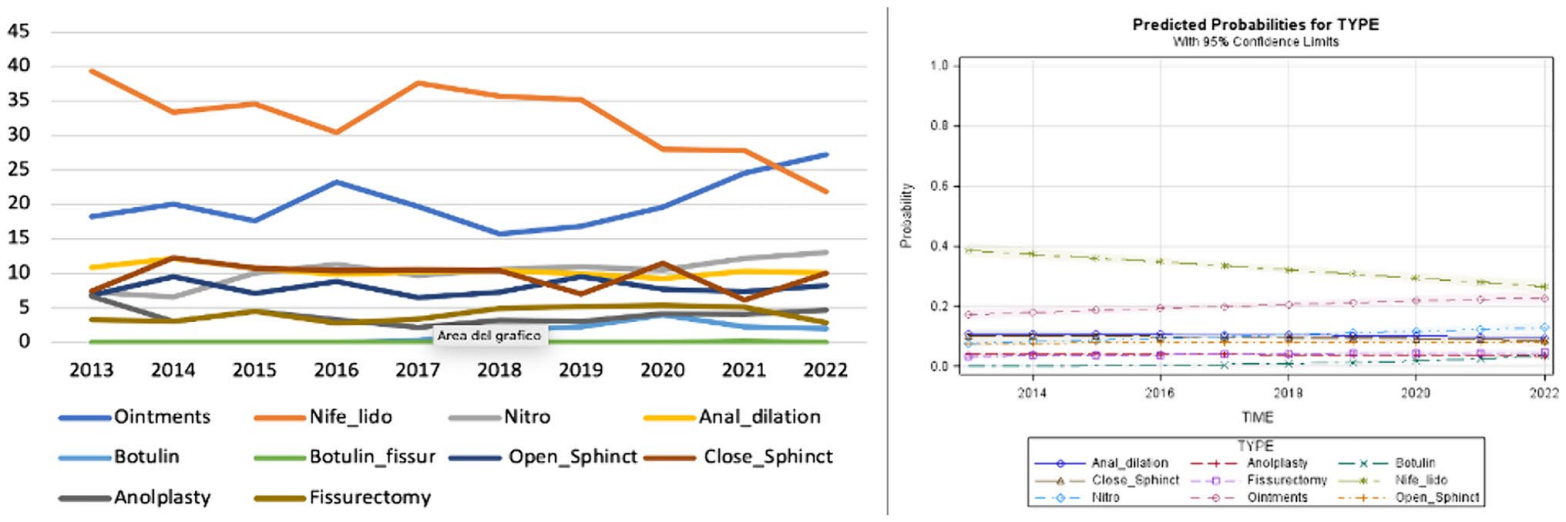
Trend of the different medical and surgical treatments over time and predicted probability model for scenario E (hypertonic internal anal sphincter)

This scenario, the most common for chronic AF, included 6181 patients. Overall, medical treatment was administered in 63% of subjects, with anal dilation or BTX injection carried out in 10.4% and 1.2%, respectively. Surgery was performed in 25.4% of patients. Nifedipine with lidocaine was the most administered ointment [2018 (32.6%) patients], followed by glycerin film-forming ointments (20.2%) and nitroglycerin (10.2%). Internal sphincterotomy was the most performed surgery, with the open technique in 488 (7.9%) patients and closed technique in 594 (9.6%) patients. Surgical treatments remained consistently performed over time according to the predicted probability model, while a decreasing trend was observed for nifedipine with lidocaine among medical treatments (Fig. 5).

### Scenario F—hypotonic anal sphincter

Out of 890 patients recruited, 556 (62.5%) were treated conservatively with glycerin film-forming ointments (31.2%), nifedipine with lidocaine (28.9%), and nitroglycerin (2.3%). Fifty (5.6%) patients underwent anal dilation. Fissurectomy [141 (15.8%) subjects] and anoplasty [132 (14.8%) subjects] were mostly performed, while internal sphincterotomy (open) was carried out in 11 patients.

**Fig. 6:**
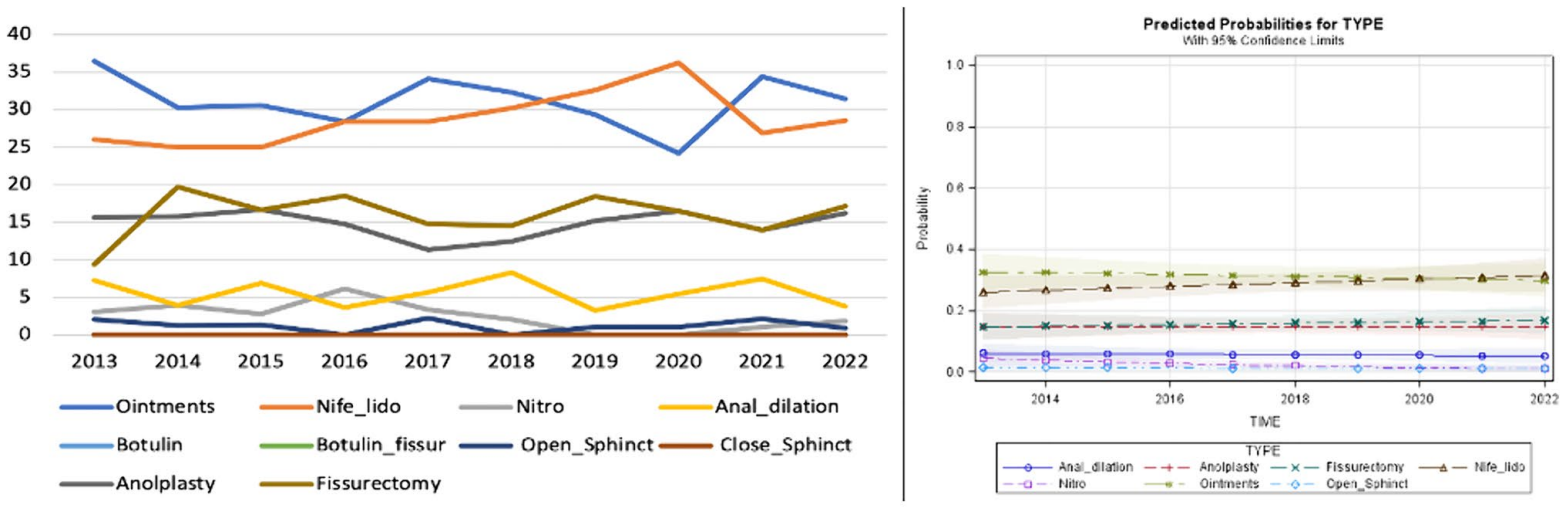
Trend of the different medical and surgical treatments over time and predicted probability model for scenario F (hypotonic anal sphincter)

An increasing trend of the nifedipine curve was observed according to the predicted probability model (Fig. 6).

## Discussion

Our study offers a comprehensive view of the evolving strategies in managing AF over a decade. The complexities inherent in treating this condition, including factors such as symptom duration, anal tone, surgeon preferences, and post-operative outcomes significantly influence treatment decisions.

While high-quality randomized evidence remains scarce [6], conservative approaches remain the cornerstone of management for AF.

The choice of proposing different scenarios according to anal tone is confirmed by the questionnaire reports, being anal tone the most important factor guiding the treatment choice of 84.3% of the responders.

Indeed, our study revealed a substantial utilization of conservative management, for both acute AF (66–75%) and chronic ones (63–67%). Moreover, the utilization rates of medical treatments exhibited nuanced shifts over time, with calcium channel blockers (particularly nifedipine with lidocaine) emerging as preferred options, aligning with evidence suggesting their efficacy in chronic AF healing [7].

Additionally, anal dilation, primarily through stretching rather than cutting of the internal anal sphincter, has gained popularity, with procedures such as controlled anal dilation showing potential equivalence to sphincterotomy in fissure healing, with minimal risk of incontinence [8]. Traditionally, this has been done by insertion of fingers into the anus, but more recently dilators have been used, which may be less traumatic [9]. Anal dilation was reported in 10–15% of patients across all scenarios except those with hypotonic sphincters.

Despite BTX injection’s proven efficacy (cure rate of 60–80%) and reduced risk of incontinence [10], its utilization in our study remained low probably due to national off-label considerations and the lack of consensus on optimal dosing and injection sites [11]. However, recent findings suggest promising avenues for optimizing chronic AF management by exploring higher doses and anterior injection sites of BTX.

Surgical intervention becomes necessary when conservative measures fail to provide relief, with fissurectomy and anoplasty emerging as prominent interventions across various clinical scenarios, probably due to the postoperative incontinence risk of sphincterotomy. Current data on postoperative incontinence after fissurectomy are heterogeneous with some reports showing intact continence and others up to 18% of minor incontincence [12–14]. Notably, they were the sole approach in scenario C (acute AF with a hypotonic sphincter). Closed sphincterotomy, preferred in specific scenarios (B and E, acute and chronic AF with hypertonic internal sphincter, respectively), offers a safer alternative to open sphincterotomy [15], with lower risks of delayed healing and postoperative complications [16].

Moreover, the study highlights the importance of considering patient-centered factors in treatment decision-making, with symptom duration and anal tone emerging as primary considerations among clinicians. However, it is noteworthy that patient preferences are less frequently factored into treatment decisions, suggesting an area for potential improvement in clinical practice.

Limitations of our study include its retrospective nature and reliance on self-reported data, which may introduce biases. Furthermore, the study’s generalizability may be limited to the Italian population. Additionally, our study lacks outcome data for the various treatment techniques evaluated, does not provide the percentage of patients undergoing a shift to surgical interventions, and does not account for potential variations in treatment practices across different regions or healthcare systems. These limitations should be considered when interpreting the findings of our study and warrant further investigation in future research endeavors.

In conclusion, our multicenter study provides valuable insights into the evolving landscape of AF management, highlighting the interplay between conservative and surgical approaches over a 10 year period. Future research endeavors should focus on prospectively evaluating the long-term outcomes of different treatment modalities and incorporating patient-reported outcomes to further optimize clinical decision-making in this challenging condition.

## Supplementary Information

Below is the link to the electronic supplementary material.

Supplementary file1 (DOCX 1266 KB)

Supplementary file2 (DOCX 16 KB)

## Data Availability

No datasets were generated or analyzed during the current study.
