# Supplementary material for: Temporal trends and treatment patterns in anal fissure management: insights from a multicenter study in Italy
Source: Tech Coloproctol. 2024 Oct 4;28(1):139. doi: 10.1007/s10151-024-03011-4 (PMC11452494; doi:10.1007/s10151-024-03011-4)

**Appendix 1.** Preliminary questionnaire

**Question 1**


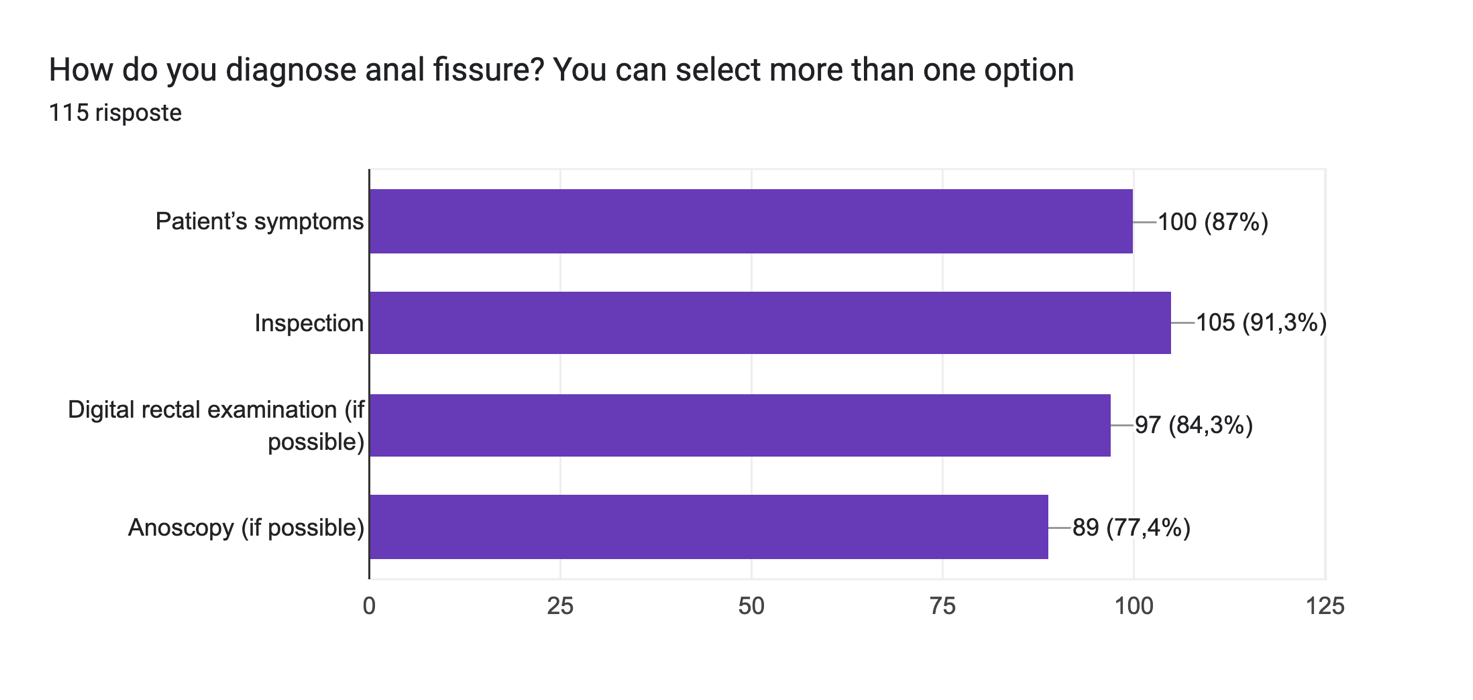


**Question 2**


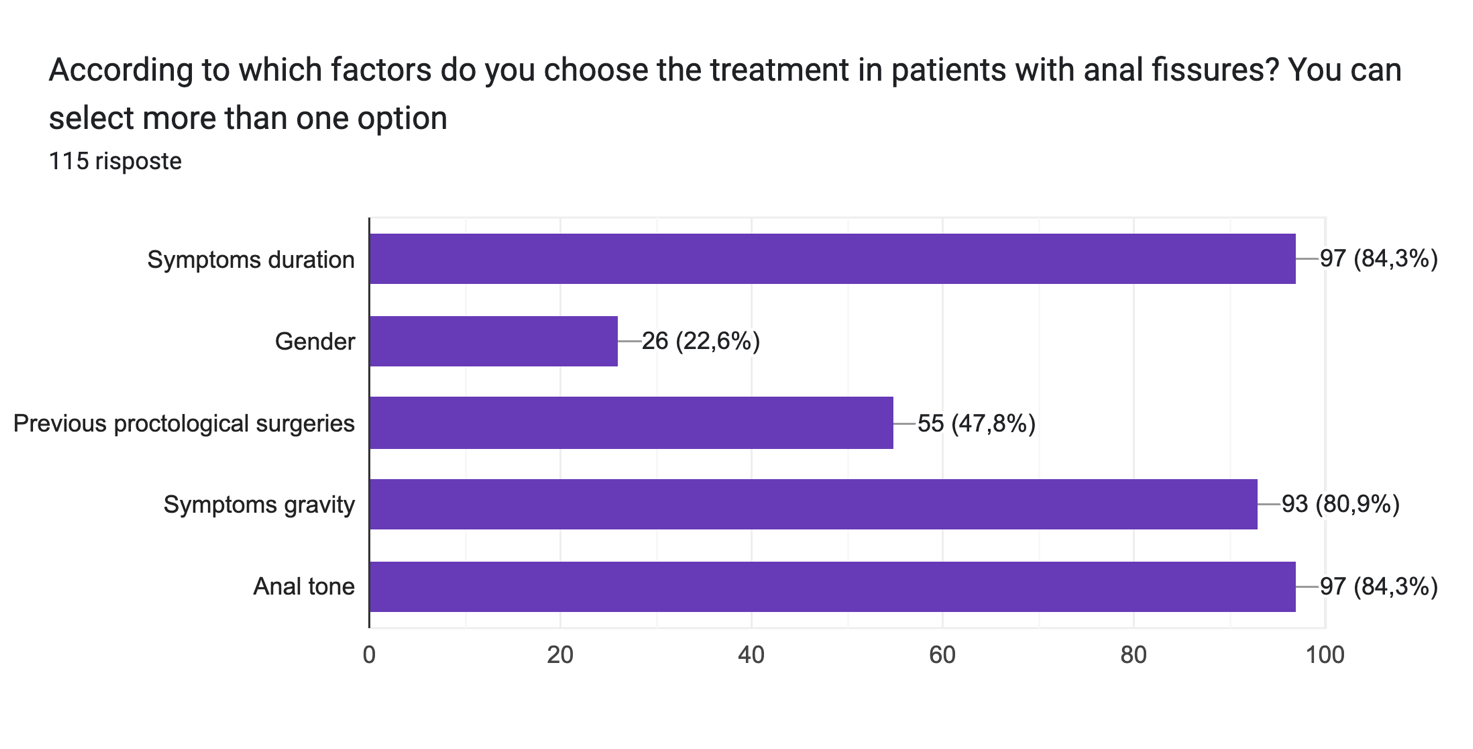


**Question 3**


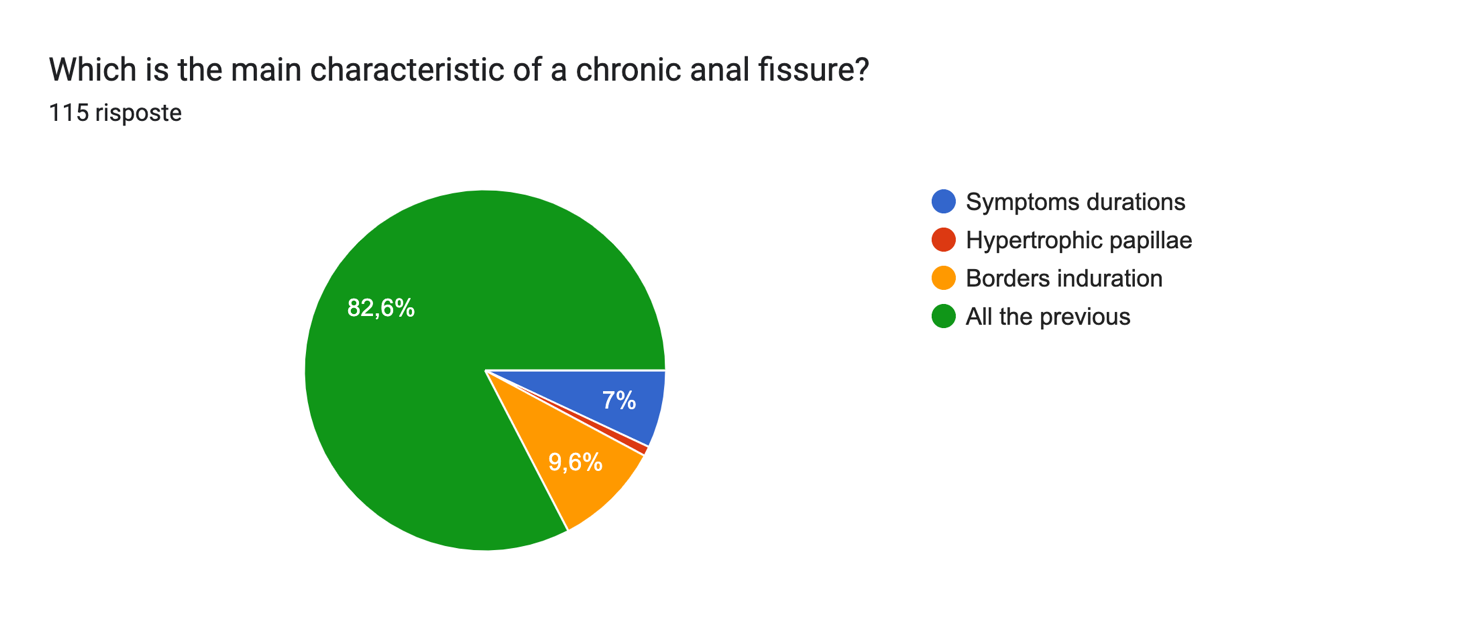


**Question 4**


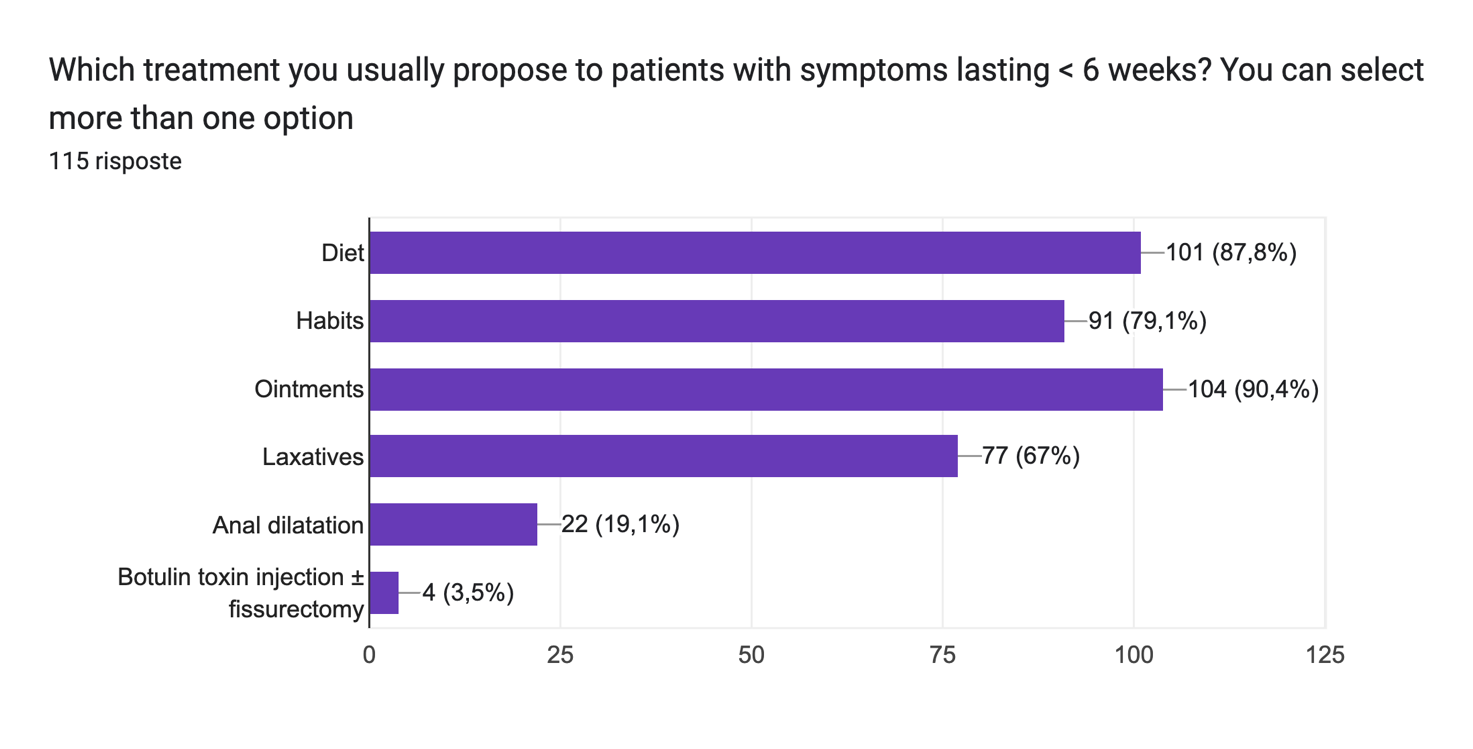


**Question 5**


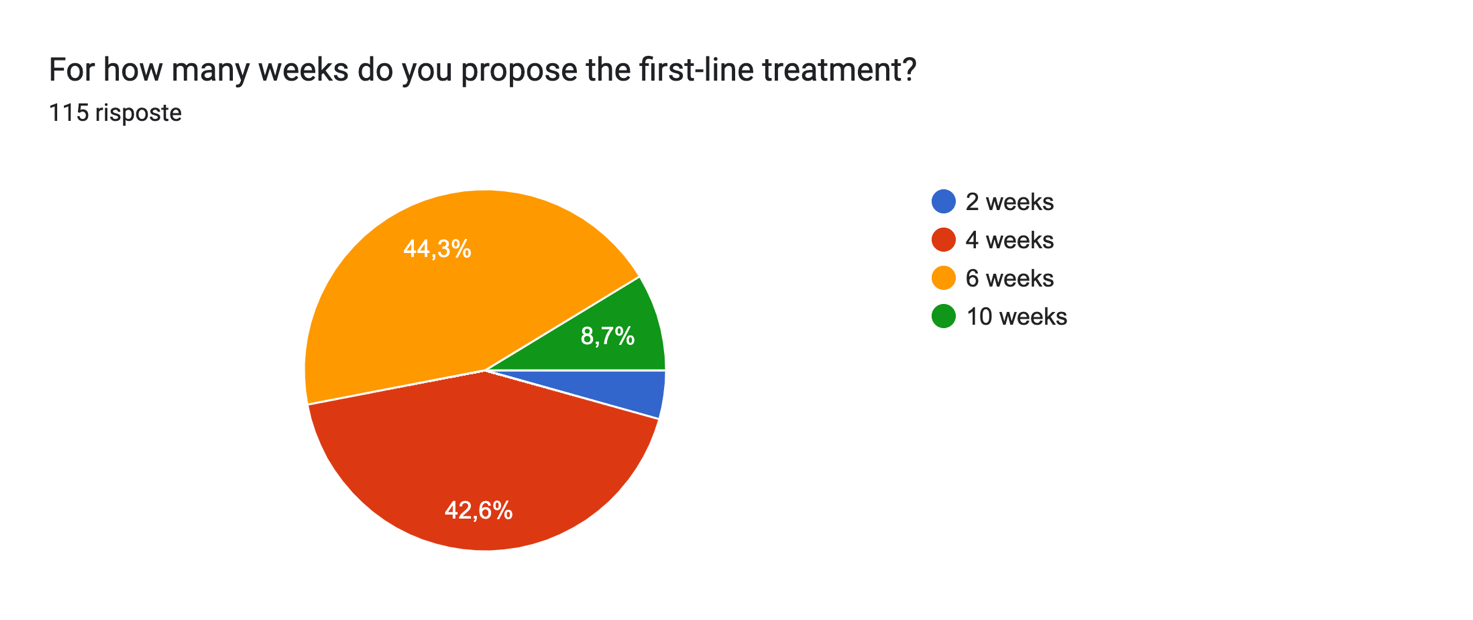


**Question 6**


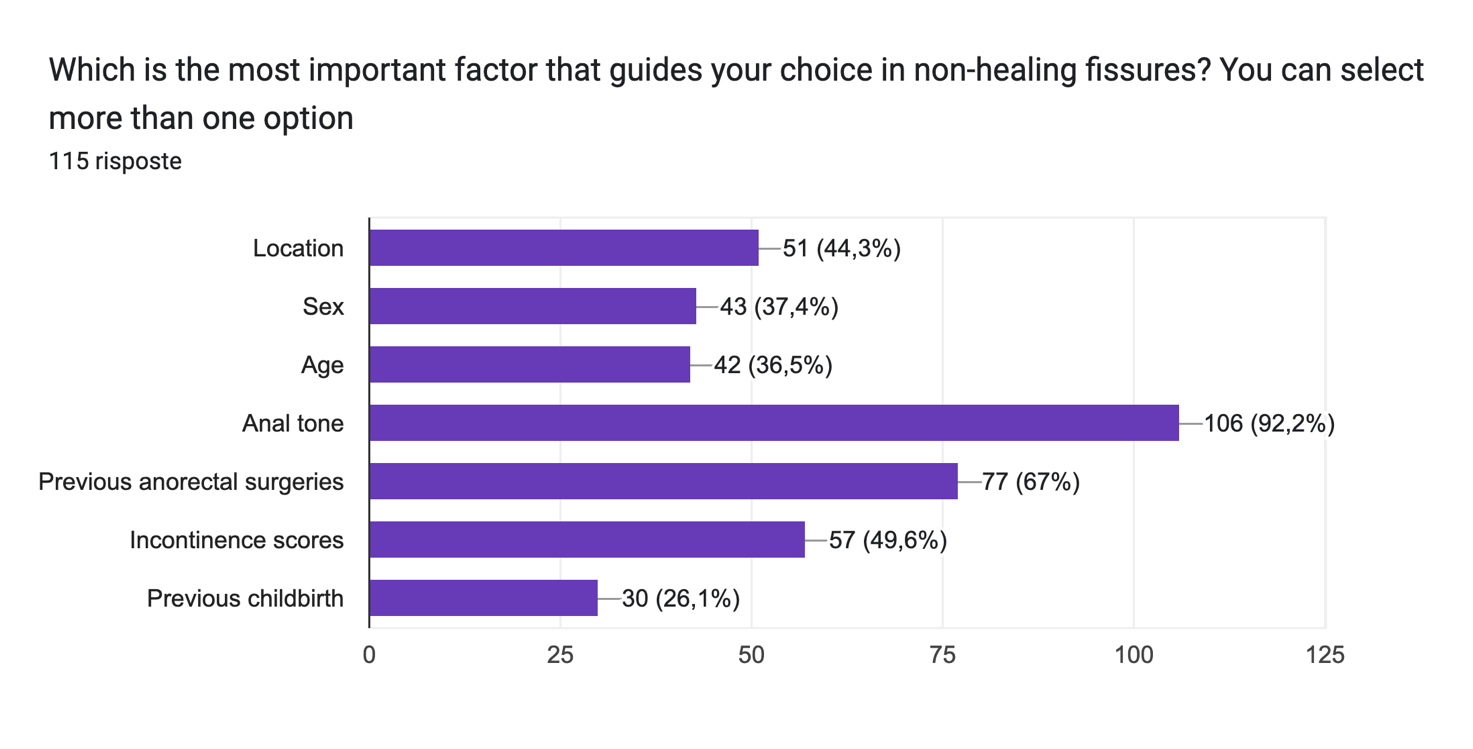


**Question 7**


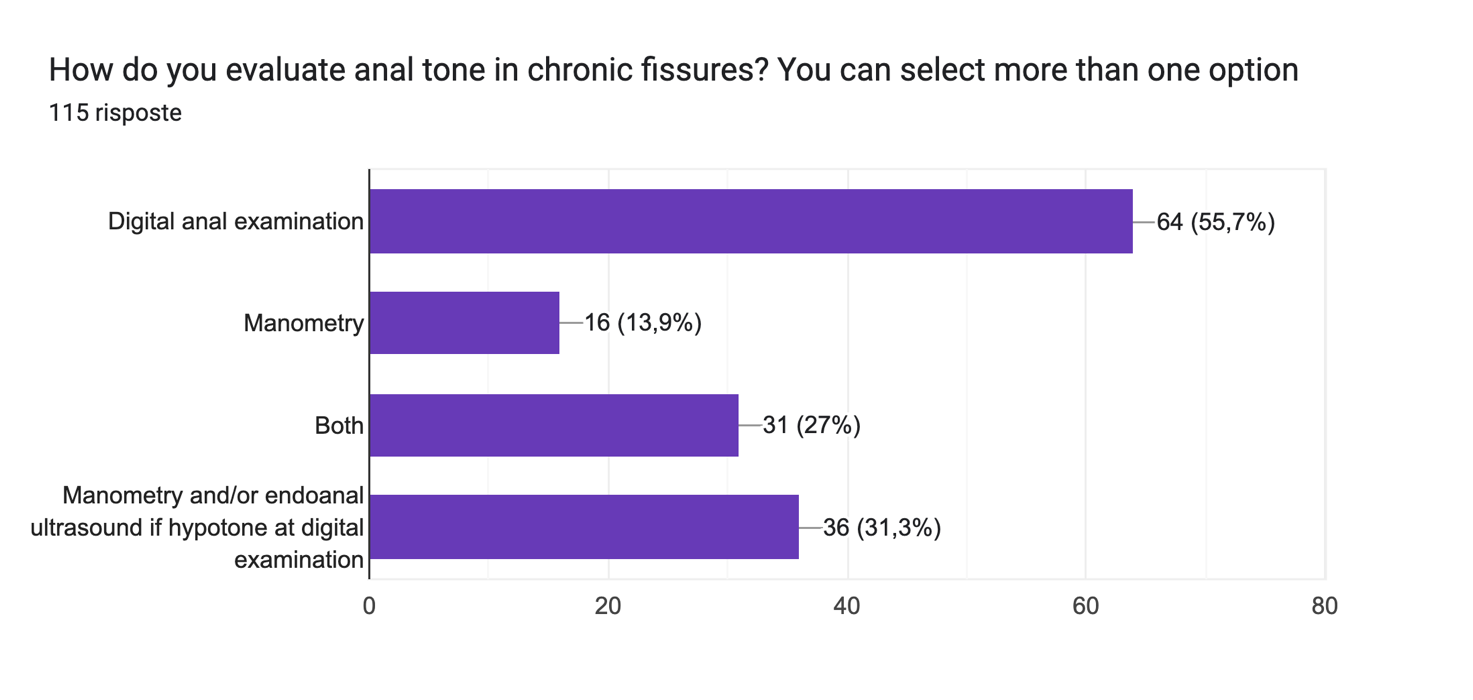


**Question 8**


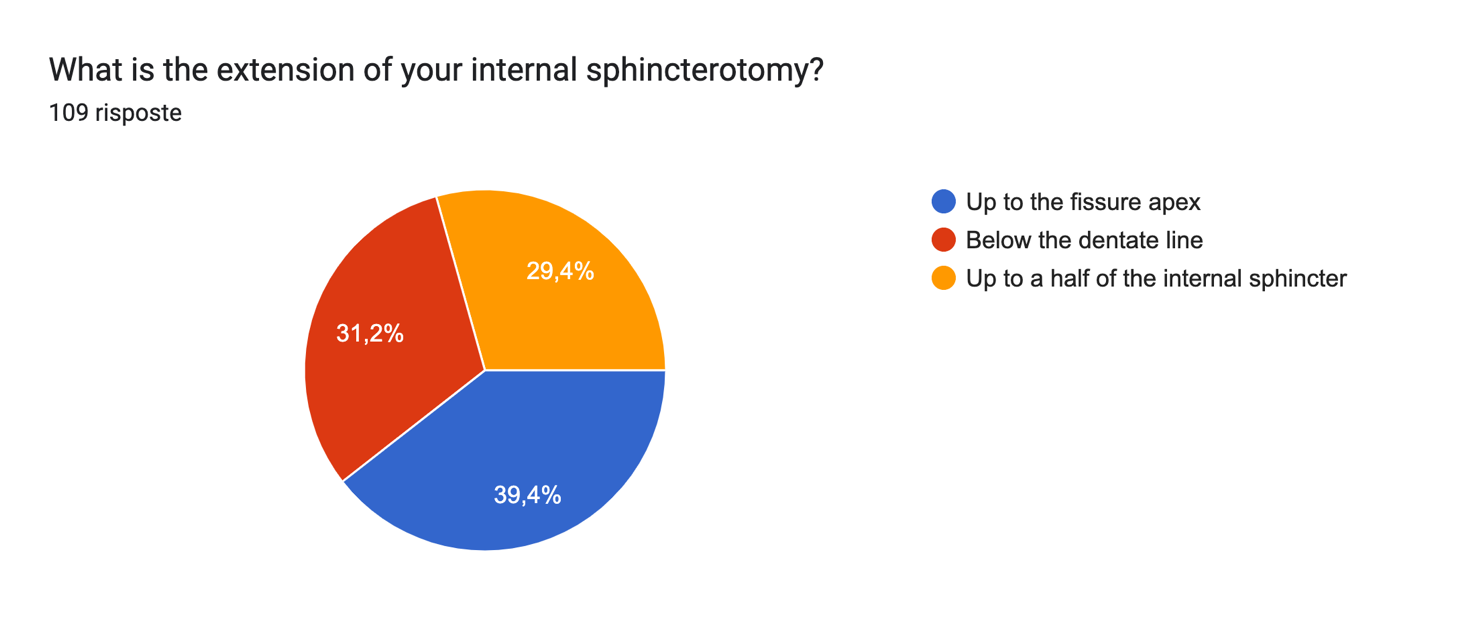


**Question 9**


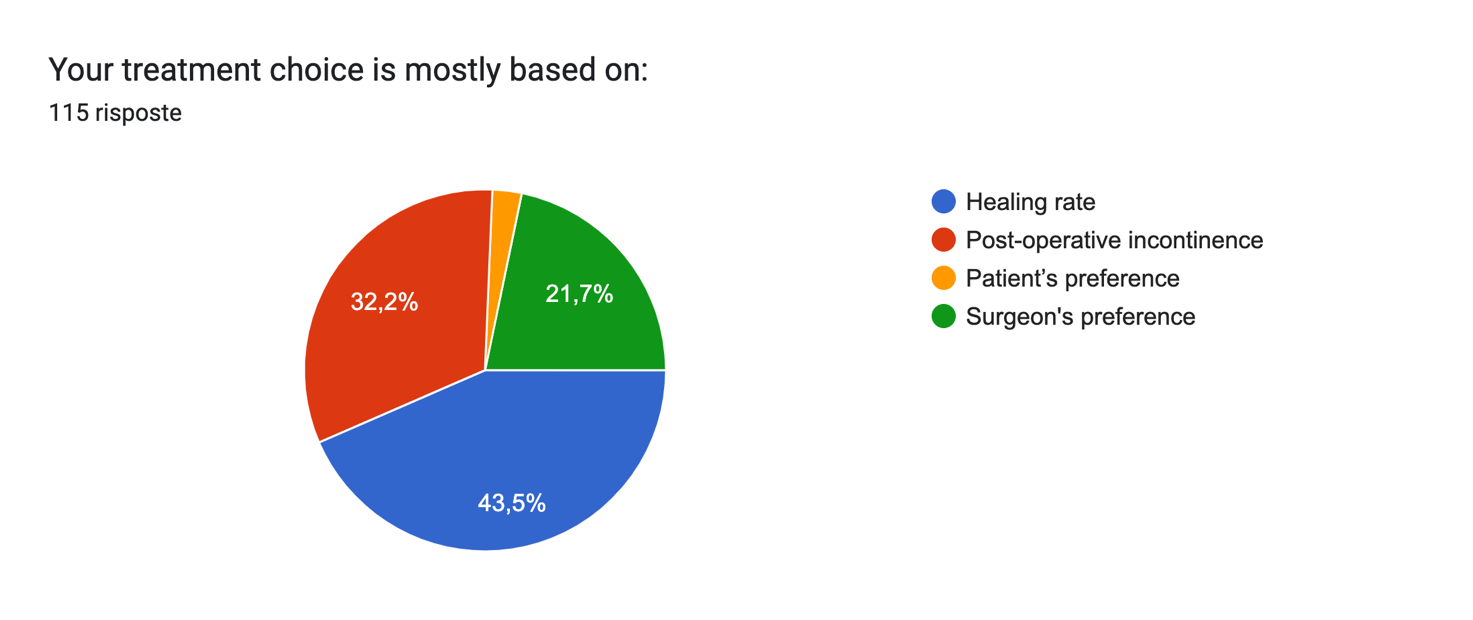


**Question 10**


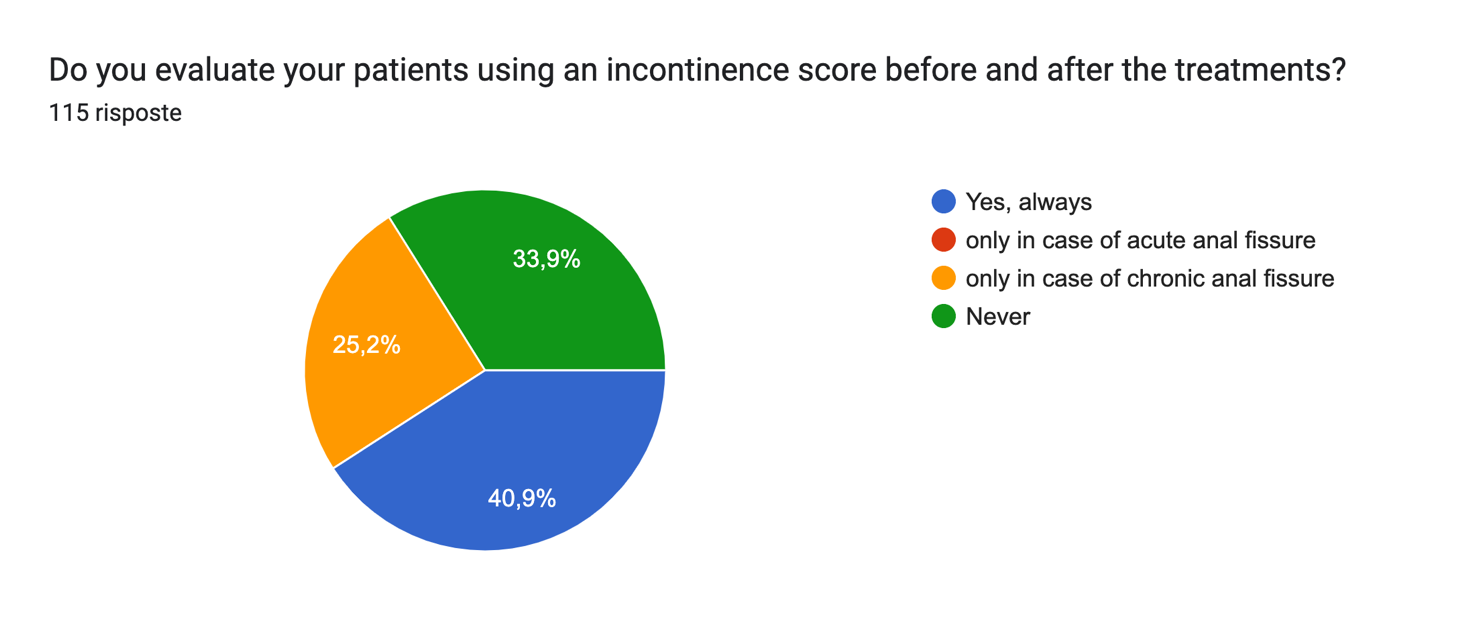


**Question 11**


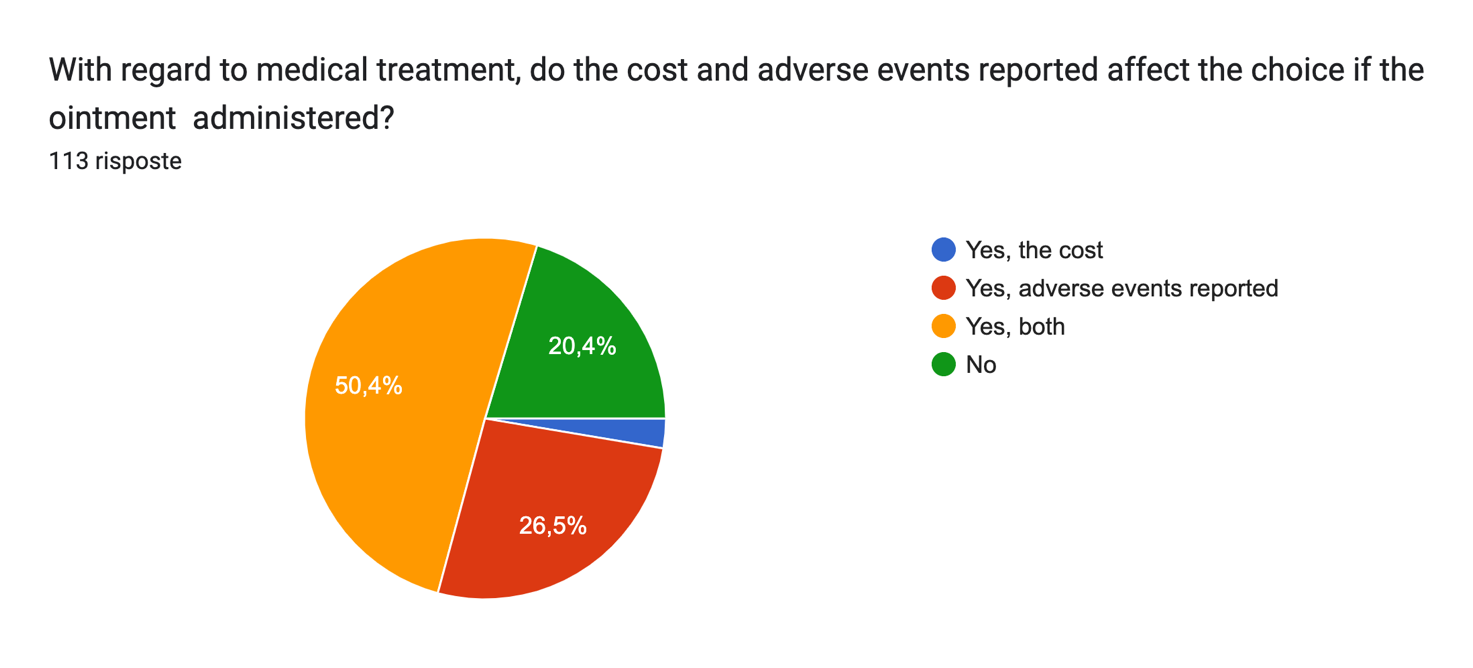

Supplement: Supplementary file 1 — Supplementary file1 (DOCX 1266 KB) [file 10151_2024_3011_MOESM1_ESM.docx]
