# Supplementary material for: Temporal trends and treatment patterns in anal fissure management: insights from a multicenter study in Italy
Source: Tech Coloproctol. 2024 Oct 4;28(1):139. doi: 10.1007/s10151-024-03011-4 (PMC11452494; doi:10.1007/s10151-024-03011-4)
Supplement: Supplementary file 2 — Supplementary file2 (DOCX 16 KB) [file 10151_2024_3011_MOESM2_ESM.docx]

**Appendix A**

SICCR Anal Fissure Group - Collaborators to be indexed:

Ambrosini Fabio: ambrofabio88@tiscali.it (PO Popoli ASL Pescara), Annicchiarico Alfredo: alfredoanni@gmail.com (Università di Parma), Antonacci Nicola: nicola.antonacci@gmail.com (AUSL Romagna Ospedale Bufalini Cesena SSD Degenza Breve Chirurgica), Ascari Francesca: francescalianaascari@gmail.com (Ospedale di Carpi AUSL Modena), Ascenzi Pasquale: p.ascenzi@ausl.bologna.it (Chirurgia B - ospedale maggiore Bologna), Balla Andrea: andrea.balla@gmail.com (IRRCS San Raffaele Scientific Institute, Milan, Italy), Barugola Giuliano: giuliano.barugola@sacrocuore.it (IRCCS Sacro Cuore Don Calabria di Negrar di Valpolicella (VR), Basso Luigi: luigi.basso@uniroma1.it (Sapienza University of Rome), Beati Claudio: claudiobeati@yahoo.com (Columbus Clinic Center of Milan), Bellato Vittoria: vittoria.bellato@gmail.com (Minimally Invasive Surgery Unit, Tor Vergata University Hospital), Bellocchia Alex Bruno: alexbellocchia@gmail.com (Ospedale Maria Vittoria, Torino), Benatti Emanuela: emanuelabenatti@libero.it (ASL 4 Liguria), Bertoli Paolo: paolobertoli80@gmail.com (Azienda Ospedaliera Papa Giovanni XXIII Bergamo), Bonomo Luca Domenico: luca.bonomo@live.it (General Surgery Unit - Rivoli Hospital),Bottini Corrado: scrivi@corradobottini.com (Humanitas materdomini Castellanza), Bracchitta Salvatore: dott.salvatorebracchitta@gmail.com (UCP Ragusa), Cafaro Danilo: danilo.cafaro@tiscalinet.it (UCP Tropea Servizio di chirurgia Proctologica), Calussi Marco: calussi.marco@gmail.com (Ospedale Santa Maria Annunziata, Firenze), Caminati Filippo: filippo.caminati@hotmail.it (SOSD Proctologia USL Toscana Centro Firenze), Candilio Giuseppe: g.candilio@alice.it (Pineta Grande Hospital Castelvolturno), Cantarella Salvatore: salvatore.cantarella@gmail.com (Ospedale di Melzo), Carbone Fabio: fa.carbone87@gmail.com (European Institute of Oncology IRCCS, Milan, Italy), Carini Stefano: stefano.carini@asst-settelaghi.it (SC Chirurgia Generale Luino-Varese), Carrino Francesco: francescocarrino@libero.it (P.O. "Maresca"), Casoni Pattacini Gianmaria: gianmaria.casonipattacini@gmail.com (Baggiovara Civil Hospital), Cestaro Giovanni: giovacestaro@gmail.com (ASST VALLE OLONA - Ospedale di Gallarate), Chessa Antonella: chant.ssa@libero.it (Chirurgia Generale usltoscanasudest), Ciferri Enrico: enrico.ciferri@galliera.it (Ospedale Galliera), Clementi Marco: marco.clementi@univaq.it (Ospedale San Salvatore, Università’ degli Studi di L’Aquila), Coco Claudio: claudio.coco@unicatt.it (Università Cattolica del Sacro Cuore - Fondazione Policlinico Universitario A.Gemelli - IRCCS), Cocorullo Gianfranco: gianfranco.cocorullo@unipa.it (AOU Policlinico “Paolo Giaccone, Palermo), Colombo Francesco: francesco.colombo@asst-fbf-sacco.it (L.Sacco University Hospital- Milano), Comba Andrea: andreacomba@gmail.com (AOU Città di Salute e fu Scirnza di Torino), Conti Luigi: dr.luigiconti@gmail.com (Casa di cura privata Piacenza), Cracco Nicola: nicracco2@gmail.com (General Surgery IRCSS Sacro Cuore Don Calabria Hospital Negrar Vr), Cravero Francesca: francesca.cravero@gmail.com (S.C. Chirurgia Generale Ospedale S.Spirito Casale M.to (AL), Crea Nicola: creanico@gmail.com (U.o Chirurgia Generale Asst Franciacorta ), Crescenti Fabio: fabiocre@hotmail.com (Ospedale di Patti), Cuccomarino Salvatore : salvatore.cuccomarino@gmail.com (Clínica Santa Caterina da Siena Torino), D'Acapito Fabrizio:, fabrizioda@gmail.com (General and Oncologic Surgery ,Morgagni-Pierantoni Hospital, Forlì), D'Onghia Giuliano: giuliano.donghia@uniroma1.it (Sapienza - Università di Roma), De Rosa Michele: michele.derosa@nhs.net (“San Giovanni Battista” Hospital, Foligno (PG), Di Pietrantonio Daniela: danidipietrantonio@gmail.com (Ospedale Morgagni-Pierantoni), Dodi Giuseppe: giuseppe.dodi@unipd.it (Centro Pelvi Padova), Ferrario Luca: luca.ferrario05@gmail.com (Department of General Surgery, Department of Biomedical and Clinical Sciences "Luigi Sacco", "Luigi Sacco" University Hospital, Università degli Studi di Milano, 20157 Milan, Italy), Fontana Tommaso: tommasofontana2@virgilio.it (P.O. “Vittorio Emanuele” Gela), Foti Nicola: nicolafoti@hotmail.com (Ospedale "Andosilla" Civita Castellana ASL/VT), Geremia Carmelo: dott.carmelogeremia@libero.it (UCP MESSINA E REGGIO CALABRIA), Giannini Ivana: ivanagi83@yahoo.it (UOS Day Service Chirurgico Polispecialistico-Servizio di Proctologia. Ospedale Fallacara (ASL Bari), Giordano Pasquale: p.giordano@londoncolorectal.org (Royal London Hospital ), Giuliani Antonio: giuldoc@hotmail.com (“San Giuseppe Moscati@ Hospital, Aversa ), Guaitoli Eleonora: eleonoraguaitoli@gmail.com (Presidio Ospedaliero Valle d’Itria), Iachetta Roberto Paolo: rpiachetta@gmail.com (Ospedale Sassuolo), Laforgia Rita: ritalaforgia@hotmail.it (UCP Bari 2 - policlinico di Bari ), Lantone Giuliano: giuliano.lantone@gmail.com (Ospedale "Lorenzo Bonomo" Andria), Lemma Maria: lemma.maria@yahoo.it (Cernusco sul Naviglio), Lisi Giorgio: giolimas06@yahoo.it (Sant’Eugenio Hospital), Lobascio Pierluigi: pierluigilobascio@gmail.com (UCP Bari 2), Lovisetto Federico: fedelovi@yahoo.com (ASL VCO), Lucci Enrico: lucci.enr@tiscali.it (AUSL della Romagna - Ospedale Morgagni Pierantoni Forlì), Madeddu Francesco: framadeddu@gmail.com (Ospedale “C.T.O.” Iglesias (SU), Magnani Costantino: costantinomagnani@gmail.com (Ausl Imola), Manzo Carlo Alberto: carlo.manzo@unimi.it (Colorectal surgery unit, Chelsea and Westminster Hospital, London, UK), Mascali Davide: davide.mascali@hotmail.it (Garibaldi ARNAS Catania), Merlini David: dott.merlini@gmail.com (ASST-RHODENSE - Ospedale di Garbagnate Milanese), Milito Giovanni: giovanni.milito@gmail.com (Paideia international hospital ), Miro Antonio: antoniomiro2@gmail.com (PO Umberto I Nocera Inferiore ASL SA), Moggia Elisabetta: elisabetta.moggia@yahoo.com (Rivoli Hospital, ASLTO3), Monaci Iacopo: iacopo.monaci@uslcentro.toscana.it (Ospedale Santa Maria Annunziata Firenze), Mozzon Marta: Marta.mozzon@asufc.sanita.fvg.it (ASUFC Udine), Navarra Luca: dott.navarra@gmail.com (Ospedale di Popoli (PE), Oggianu Angelo: angelo.oggianu@libero.it (Ospedale di San Gavino Monreale), Orlandi Simone: simoneorlandi@gmail.com (Department of Gastroenterology and Endoscopy, Sacro Cuore Don Calabria Hospital, Negrar, Verona, Italy), Pafundi Donato Paolo: docpafundi@hotmail.it (Policlinico A. Gemelli), Palumbo Alessio: ilchirurgo@hotmail.it (Chirurgia3 AORN Cardarelli Napoli), Passaro Umberto: umberto.passaro@aslroma2.it (UCP S. Eugenio Roma), Pata Francesco: francesco.pata@gmail.com (Department of Pharmacy, Health and Nutritional Sciences, University of Calabria, Rende, Italy), Pecorella Giuseppe: gpecorel@unict.it (Clinica Morgagni catania), Pedrazzani Corrado: corrado.pedrazzani@univr.it (University of Verona), Piccolo Davide: davide_piccolo@yahoo.it (Ospedale di Piacenza), Poli Giulia: gulia.poli@gmail.com (ASL Nord Ovest Toscana, Villamarina Hospital, Piombino (Li), Rinaldi Marcella: marcella.rinaldi@uniba.it (University “Aldo Moro” of Bari, Bari, Italy), Ripetti Valter: v.ripetti@unicampus.it (UCP Roma2), Rizzo Salvatore: salvatorerizzo.ch@gmail.com (Ospedale di Cavalese APSS TN), Rocco Giuseppe: giusepperocco12@gmail.com (Ospedale Morgagni-Pierantoni di Forlì), Romano Francesco Maria: francescomaria.romano@unicampania.it (AOU Vanvitelli), Sacco Michele: michelesacco1985@gmail.com (PO Camberlingo Francavilla Fontana), Sallustio Pierluca Nicola Massimo: lucustio@hotmail.com (Policlinico di Bari), Santoro Giulio Aniello: giulioaniello.santoro@aulss2.veneto.it (Ospedale Regionale di Treviso, AULSS2 Marca Trevigiana), Saroglia Giuliano: giusaroglia@gmail.com (villa maria pia hospital), Scotto Bruno: dr.brunoscotto@gmail.com (Ramazzini Hospital Carpi - AUSL Modena), Selvaggi Lucio: lucio.selvaggi@gmail.com (Università della Campania Luigi Vanvitelli), Silvestri Vania: vaniasilvestri83@gmail.com (Chirurgia Generale, PO SS Annunziata Sulmona), Soldini Gabriele: dott.g.soldini@gmail.com (Ospedale Fatebenefratelli Erba), Tamburini Andrea Marco: tamburini.andreamarco@hsr.it (Chirurgia Gastroenterologica IRCCS Ospedale San Raffaele), Tamini Nicolò: nicolo.tamini@gmail.com (IRCCS Ospedale San Gerardo Monza), Tanda Cinzia: cinzia.tanda@gmail.com (Casa di Cura "Madonna del Rimedio"), Terrosu Giovanni: giovanni.terrosu@uniud.it (Clinica Chirurgica - ASUFC Az Sanitaria Universitaria Friuli Centrale), Testa Alessandro: testaalessandro@fastwebnet.it (Ospedale San Pietro FBF), Tomasicchio Giovanni: giovannitomasicchio92@gmail.com (Università degli studi di Bari), Turati Luca: dott.luca.turati@gmail.com (Ospedale Pesenti-Fenaroli di Alzano Lombardo - ASST Bergamo Est), Ursino Natale: natale.ursino75@gmail.com( UCP Città di lLcce Hospital), Vannelli Alberto: info@albertovannelli.it (Ospedale Valduce), Viola Gabriele: gabrieleviola@yahoo.com (Ospedale dell'angelo - Mestre), Violante Tommaso: tommyviolante@gmail.com (Unibo Alma Mater Studiorum), Zigiotto Daniele: daniele.zigiotto@gmail.com (Ospedale Fracastoro, San Bonifacio (VR)
